# Tobacco Denormalization Indicator in the Prevalence of Positive Smoker Identity and Its Associated Factors

**DOI:** 10.3390/ijerph17072363

**Published:** 2020-03-31

**Authors:** Mohd Hanief Ahmad, Mohd Ismail Ibrahim, Azriani Ab Rahman, Kamarul Imran Musa, Faridah Mohd Zain, Rehanah Mohd Zain, Ruhaya Hasan, Noraryana Hassan, Imran Ahmad, Nur Suhaila Idris

**Affiliations:** 1 Department of Community Medicine, School of Medical Sciences, Universiti Sains Malaysia, Kubang Kerian, 16150 Kelantan, Malaysia; drhanief@moh.gov.my (M.H.A.); azriani@usm.my (A.A.R.); drkamarul@usm.my (K.I.M.); 2 Department of Fa and ly Medicine, Hospital Universiti Sains Malaysia, School of Medical Sciences, Universiti Sains Malaysia, Kubang Kerian, 16150 Kelantan, Malaysia; faridahz@usm.my (F.M.Z.); profimran@usm.my (I.A.); nursuhaila@usm.my (N.S.I.); 3 School of Health Sciences, Universiti Sains Malaysia, Kubang Kerian, 16150 Kelantan, Malaysia; rehanah@usm.my; 4 School of Dental Sciences, Universiti Sains Malaysia, Kubang Kerian, 16150 Kelantan, Malaysia; ruhaya@usm.my; 5 FCTC and Tobacco Control Unit, Disease Control Division (NCD), Ministry of Health Malaysia, 62590 Putrajaya, Malaysia; noraryana@moh.gov.my

**Keywords:** positive smoker identity, prevalence, associated factors, PSmoQi, tobacco denormalization

## Abstract

Positive smoker identity (PSI) is a construct that evaluates the degree of smokers’ positive thoughts, images and feeling about smoking behavior and culture. PSI encompasses the indicators related to tobacco denormalization strategy, which is one of the four WHO tobacco endgame strategies. PSmoQi is a newly validated instrument which could reliably assess PSI. This study’s objectives were to determine the prevalence of positive smoker identity and its associated factors using PSmoQi. A sample of 253 smokers from government agencies in Kota Bharu City, Malaysia were recruited using invitation letters sent to their head of agencies. Data collection was done in a briefing session voluntary attended by the smokers. Factors associated with PSI were analyzed using Multiple Logistic Regression. The prevalence of smokers with positive smoker identity was 72.3%. Factors associated with positive smoker identity were older age (Adjusted Odds ratio; AOR: 1.042; 95% confident interval; CI: 1.004, 1.081); *p* = 0.028), higher smoking self-concept scale Malay version (SSCS-M) score (AOR: 1.216; 95% CI: 1.112, 1.329; *p* < 0.001), higher heaviness index (AOR: 1.002; 95% CI: 1.001, 1.004; *p* = 0.011) and lower educational attainment (AOR: 0.458; 95% CI: 0.233, 0.900; *p* = 0.024). This study shows a high prevalence of PSI among smokers from government agencies in Kota Bharu City. Factors such as age, SSCS-M score, heaviness index and educational attainment influenced the level of positive smoker identity in a smoker. The finding would contribute an evidentiary guideline in screening smokers for smoking cessation clinic enrollment to achieve the best interventional outcome, as well as it would provide an objective indicator for tobacco denormalization status in a population.

## 1. Introduction

### 1.1. Identity Construct in PRIME Theory for Smoking Behavior

**Figure 1:**
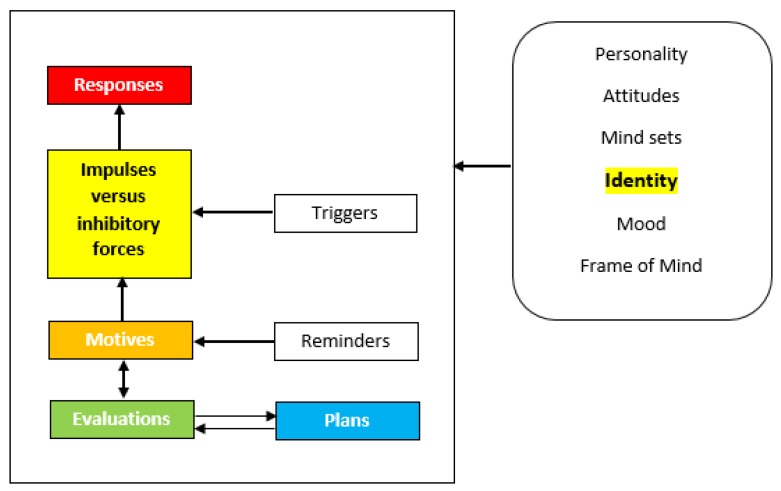
Schematic illustration of five stages in West’s PRIME Theory [2]. Chances for impacts between the stages are demonstrated by their being next to each other in the illustration. For instance, motives could only affect responses through impulses and evaluations could only impact upon responses through motives and then impulses. Plans contribute a framework to our responses but could only affect them through motives and evaluations functioning in the moment when they are to be implemented.

In the interest of cigarette smoking cessation, positive smoker identity construct was identified to have some influence in the cessation success [1]. Positive smoker identity—sometimes called the smoker identity—is one’s positive feelings attached to the identity as a smoker. It includes positive thoughts and positive images of a person’s cigarette smoking act and his or her positive feeling about smoking. Positive smoker identity incorporates thoughts of belonging to the smoker category or label. For example, those smokers who do not have positive smoker identity may incline towards being labeled as non-smoker category, rather than the smoker category. West’s PRIME Theory illustrates the intricacy of why people persist or cease smoking according to five stages of motivational structure including responses, impulses, motives, evaluations and plans [2]. Smoker identity is one element of the internal environments which influences these 5 stages (Figure 1). The identity construct would directly have impacts on all stages which would then lead to the response, either to continue smoking or to stop. Chances for impacts between the stages are demonstrated by their being next to each other. For instance, motives could only affect responses through impulses and evaluations could only impact upon responses through motives and then impulses. Plans contribute a framework to our responses but could only affect them through motives and evaluations functioning in the moment when they are to be implemented.

Knowledge of the degree of positive smoker identity in a smoker would provide a considerable aid in smoking cessation clinic whereby smokers who have low degree of PSI could be prioritized more than those who have higher degree of PSI. Nevertheless, a recent study suggests that PSI could become a good indicator for denormalization of cigarette smoking should it be tested in a larger population such as in a community, in a state or in a country [3].

### 1.2. Positive Smoker Identity as Denormalization Indicator

PSmoQi© was developed and reliably demonstrated as a validated instrument in evaluating the degree of PSI in a smoker [3]. It contains 6 domains comprising contributory factors, external awareness, Identity related to smoking, superego challenge, contextual and temporal patterning and behavior in relation to smoking. These six domains can quantify the degree of PSI, based on a proposed scoring system. As recommended by the study, a larger scale study could unearth the degree of PSI in a larger population, indicating how well smoking behavior being accepted or rejected in a community, a state or a country. Each of six domains contained specific elements related to denormalization component. However, the proposed scoring system would distinguish a smoker with PSI from a smoker without PSI based on a recommended cut-off point (high score versus low score). Therefore, once the binary categorization is done for individuals, the prevalence of PSI may generally indicate the denormalization as a whole in a group of people, a society or a country.

### 1.3. Prevalence and Factors Associated with Positive Smoker Identity (PSI)

Table 1 summarized literature reviews on the prevalence of PSI (or constructs identical to PSI) and factors associated with PSI.

The strength of the study by Berg et al. [3] was contributed by their relatively large sample size (9931 participants) and multivariate analysis using binary logistic regression. They also showed that young smokers who denied being a smoker were more likely not attempting to quit smoking. Choi et al. [4] who used the term “phantom smoker” to indicate smokers with non-smoker identity found that phantom smokers smoked less in terms of amount and frequency than smokers with positive smoker identity, were more likely to smoke in social situations, especially in a bar or with friends. Levinson et al. [5] attempted to identify if there was any difference in response to a question asking whether a respondent was a “smoker” or a “social smoker”. This study’s strength was its focus in deeply scrutinizing the term “social smoker” in comparison to “occasional smoker” and non-smoker identity. Ridner et al. [6] noticed that individuals who self-described as non-smokers had the lowest current smoking rate (4.6%) when compared to individuals who self-described as smokers (97.5%). The strength of this study was their spotlight on the discordance (disagreement) between the empirical classification of whether an individual was a current smoker or a non-smoker and an individual’s self-described smoking identity. Hertel and Mermelstein [7] found that the more adolescents thought smoking was a defining aspect of who they were, the more likely their smoking escalated. Tombor et al. [1] found that positive smoker identity was more likely to be in individuals who are older, male, more nicotine dependent, have lower motivation to stop, have not made a quit attempt in the past year, enjoy smoking and consider themselves to be addicted. They also report that having a positive smoker identity independently predicted failure to make a quit attempt at six months. The same authors did another study which focused on ex-smokers who already quit smoking in the past 1 year [11]. They discovered that most people (80.3%) who quit smoking recently consider themselves as non-smokers and younger people and those who have been abstinent for longer were more likely to take on a non-smoker identity.

Most of the above studies have their own limitations such as a confined study population (Berg et al., 2009) [4], the usage of convenience sampling in subject selection (Choi et al., 2010; and Levinson et al., 2007) [5,6] and a low response rate at 18.5% (Ridner et al., 2010) [7]. However, an immense limitation of all, that is synonymous in all the previous studies on PSI, was the usage of a single yes or no question to measure the PSI construct (or construct identical to PSI). In these studies, there were lack of validated or reliable instrument used to distinguish those with PSI from those without PSI. This limitation could lead to an ascertainment bias because the instrument or tool utilized was devoid of complexity and richness in defining those with PSI.

### 1.4. Research Goals

The objective of this study was to determine the prevalence of smokers who had positive smoker identity, which was indicated by high PSmoQi score. The second objective was to identify factors associated with high PSmoQi score.

## 2. Materials and Methods

### 2.1. Sample Size Calculation

We utilized a single proportion formula in calculating sample size for the objective of determining the prevalence of smokers who had positive smoker identity, which was indicated by high PSmoQi score:

*n* = (Zα/E)^2^ × P (1 − P) (1)

Z was the value from the standard normal distribution demonstrating the confidence level (CI) that was used. Therefore, at CI of 95% (α = 0.05), the Z value was 1.96. E was the pursued margin of error or also called precision, which we took E = 0.05. P was the proportion of smokers with positive smoker identity in the smoking population. Here, we designed a study to achieve a 95% confidence interval for the unidentified population proportion (P). Because P was unknown, an approximate value of P was taken from a study [1] who found that the prevalence of positive smoker identity was 18% in their study (P = 0.18). Hence, the calculated sample size here was *n* = 226.

For the objective of determining factors associated with positive smoker identity, two proportion formula was used. Table 2 showed the sample sizes calculated using PS Software according to the factors associated with positive smoker identity found in other previous studies. Based on this objective, a total maximum of 440 working adults was supposed to be recruited for this study to allow for an expected 20% non-response rate and missing data. All in all, this number was the biggest sample size calculated among all objectives. So, we considered this number as the required sample size in the study.

### 2.2. Recruitment, Sampling and Data Collection Procedure

Letters were sent to the head of departments of all government agencies in Kota Bharu, a city with a population of about 600,000 in northeast coast of peninsular Malaysia. The letters contained the objectives of the study, the structure and planning of the research and a request for participation of their staff who smoked. A preliminary form containing ID number, name of agency and 3 questions were distributed to every staff in their department. The 3 questions/statements were: “I currently smoke”, “I occasionally smoke” and “I do not smoke at all”. The answer choices were “Yes” and “No”. Those who answered “Yes” to either question 1 or 2 were shortlisted. A sequential number was assigned to those shortlisted as a sampling frame.

According to the initial plan, simple random sampling was supposed to be done through random number assignment using SPSS software version 22 (Statistical Package for the Social Sciences, IBM Corp, Armonk, NY, USA) in conformity with the sampling frame and required sample size. However, there were only 311 smokers listed in the sampling frame. As the number in the sampling frame was below the sample size requirement, we took all 311 samples as our study sample. Those 311 smokers were all invited to attend briefing and data collection sessions where the proforma and a set of questionnaires were distributed. A questionnaire with incomplete answers would be followed up and aimed for completion. Those who were invited but did not attend the sessions were followed up at their agencies’ office. Briefing and data collections were done in their respective offices. Nevertheless, the total number of participants who completed the proforma and questionnaires was 253 samples after all the re-invitations and follow-ups.

### 2.3. Research Instruments

Our study instruments consisted of a proforma containing socio-demographic attributes, comprehensive smoking status, cessation attempts data, self-reported health condition, awareness about anti-smoking material in the media and the economic data. Also, 30-items PSmoQi© was also used whereby the respondent’s feedbacks were evaluated using a 5-point Likert scale varying from ‘strongly disagree’ to ‘strongly agree’.

### 2.4. Data Analysis

Data were entered using Excel 2016 (Microsoft, Redmond, WA, USA) and were analyzed using R software version 3.3.1 (R Foundation for Statistical Computing, Vienna, Austria, 2013). Descriptive statistics were utilized to describe the socio-demographic characteristics of subjects. Numerical data were presented as mean (SD) or median (IQR) based on their normality distribution. Categorical data were presented as frequency (percentage).

The prevalence of smokers with positive smoker identity (PSI) we calculated with the following formula:

Prevalence of PSI = Number of respondents with positive smoker identity/Total number of respondents (2)

For the objective of determining factors associated with positive smoker identity, multiple logistic regression was used. The dependent variable used in simple and multiple logistic regression was positive smoker identity status, which dichotomized smokers with or without positive smoker identity. Selection of independent variables was based on prior knowledge from extensive literature review which supported these as potential predictors for positive smoker identity. There were 28 categorical variables and 10 continuous variables selected as the independent variables. Those variables with *p*-value < 0.25 in the simple logistic regression were included in multiple logistic regression analysis. Forward selection, backward elimination and manual entry and manual forward methods were utilized to get the best model. *p*-values of < 0.05 will be considered statistically significant. The model fitness was checked using Hosmer and Lemeshow test, classification table and area under ROC curve. All assumptions of multiple logistic regression models were evaluated as well as the multi-collinearity and interaction issue.

### 2.5. Ethics Endorsement and Consent to Involve

Research and Ethical Committee of the Universiti Sains Malaysia (JEPeM) granted ethical approval for this research (USM/JEPeM/17010063) on the 30 March 2017. The study was implemented by complying with the Declaration of Helsinki. Written consents were acquired from the respondents. The participants were allowed full autonomy in the decision for taking part in this study. Their involvements in this study were entirely undertaken by free choice. They were granted freedom to decline or to quit involvement in the study whenever they wanted, without a drawback or mislaying of advantages of which they had the right to have. The study did not influence any services or treatments rightly available for them. The independent status of the data were maintained, and they would not be utilized in any performance evaluation and verdict pertaining to healthcare plan.

### 2.6. Accessibility of Documents and Data

All hardcopy and softcopy documents in this study were maintained confidential. Research documents were kept in a secured cabinet and data were protected in a password-shielded thumb-drive. The only personnel who had access to the study data were from the research team. The datasets utilized in the study were accessible by an appropriate application to the corresponding author.

## 3. Results

### 3.1. Socio-Demographic Characteristics, Smoking Behavior Data and CFA

Table 3 demonstrates the demographics of the participants. Data on smoking behavior, cigarette cessation behavior, self-reported health status and co-morbidities, their awareness towards anti-smoking campaigns, the economics of smoking and scores for all study tools are shown in Table 4.

### 3.2. The Prevalence and Factors Associated with Positive Smoker Identity

Using a cut-off level of −43 for the total PSmoQi score, the prevalence of smokers with positive smoker identity is 72.3% (95% CI: 67–78%) out of 253 respondents.

The dependent variable used in simple logistic regression and multiple logistic regression is positive smoker identity status, which dichotomizes smokers with or without positive smoker identity. The cut-off point for a smoker with a positive smoker identity is more than −43 of the total PSmoQi score, which is the most optimal cut-off point [2]. Selection of independent variables is based on prior knowledge from extensive literature review which supports these as potential predictors for positive smoker identity. Table 5 shows the simple logistic regression table and those variables which *p*-value less than 0.25 are selected.

“Ways to stop smoking” variables were not included to the model as it destructs model stability and classes balance. Ways of stop smoking which consist of variables such as “used willpower to stop”, “over the counter medication”, “friends’ help”, “sought health counselling” and “professional NRT” show some quasi-complete separation due to the way questionnaire being structured and coded. This issue is in the interest of relationship between these variables and another important variable, “number of stop attempts”. In the questionnaire, “number of stop attempts” precedes these variables, whereby zero number of attempts would definitely render the following ways to stop smoking variable extraneous. Another reason of quasi-complete separation is due to the small sample size which caused classes’ imbalance.

The variable “no. of day cigs smoked last 30 days” was combined and computed with variable “no. of cigs per day” to form another variable called smoking heaviness index. Smoking heaviness index represents how many cigarettes a smoker smoke per month. As a result of this variable fusion, the contributory variables (“no. of day cigs smoked last 30 days” and “no. of cigs per day”) were also not picked into the model to prevent multi-collinearity among variables. We also insert a categorical variable “watched stop smoking campaign”, which has 2 categories (often vs. occasional or never), into the model despite its *p*-value exceeds 0.25 (0.387). This step is carried out to generate a possibly a richer model. We consider “watched stop smoking campaign” variable as an important confounding variable based on our comprehensive literature review [12]. Therefore, the total number of variables included in the model for multiple logistic regression were 22 variables, which comprise of 14 categorical variables and 8 continuous variables.

For multiple logistic regression, we use a forward selection method, backward elimination method and then manual entry method which reveal that a number of variables remain significant (*p* > 0.05). In order to further improve the model in term of clinical parsimony and model fitness and to acquire more variables into the model, we relook into the outliers in our data which may have possibly contributed to lack of significant predictors in the model. After eliminating the outliers, we carry out a manual forward procedure. This process of inserting, re-fixing and confirming continue until it emerges that all the relevant variables were entered in the model and those variables omitted were clinically and/or statistically unimportant.

As demonstrated in Table 6, the significant factors associated with positive smoker identity in the final model were higher SSCS Score, older age, lower education attainment and higher heaviness index. These findings come out after controlling other factors such as FTND-M score, CSEQ-M score and exposure to stop smoking campaigns in multiple logistic regression.

## 4. Discussion

The 72.3% prevalence of smokers with positive smoker identity was substantial, in comparison to the other studies. Tombor et al. [1] reported a prevalence of 18.3%, a very low figure compared to ours. Whilst Berg et al. [4], Choi et al. [5] and Levinson et al. [6] observed a prevalence of 49.3%, 26.2% and 43.7% respectively. The explanation was that this study was carried out in Kelantan where the prevalence of smokers was the highest among all states in Malaysia and was definitely above the national average of 24.9%, with a total prevalence rate record of 30.2% [13]. positive smoker identity was shown to have associations with resistance to anti-tobacco messages [9], with less intention and less attempt to quit attempt [14] and with stronger nicotine dependence and lower motivation to stop smoking [1]. These findings could explain why positive smoker identity may have a strong positive relationship with the prevalence of smoking.

In contrast to our study, the studies by Berg et al., 2009, Choi et al., 2010 and Levinson et al., 2007, had only included participants among young adults in college or universities. These differences could have contributed to the dissimilar prevalences. Berg et al., 2009, demonstrated that those denying their positive smoker identity tend to be younger. And according to Tombor et al., 2013, having a positive smoker identity was associated with being older. Being older could mean a smoker’s involvement in cigarette smoking activity for a longer duration. According to Reinforcement Theory [15], people searched and recalled information that granted cognitive support for their preceding attitudes, identity and beliefs. Therefore, since the first cigarette, older smokers had been granted more time and opportunity to reinforce their belief and identity as a smoker. In addition, Increasing Persistence Hypothesis suggested that people became gradually more resistant to change throughout their lives [16]. There was also Impressionable Years Hypothesis which suggested that people were highly susceptible to behavioral change during late adolescence and early adulthood [17].

Apart from that, all of the participants in our study were males. In contrast, the participants in almost all previous studies comprised of equal distribution between males and females. In Malaysia, the male prevalence of smoking was considerably greater (43.0%) than the female prevalence (1.4%) [18] Such disparity in gender-based prevalence was not really seen in the UK and USA. In the UK, the prevalence of smoking in male and female was 19.3% and 15.3% respectively [19]. Whilst in the USA, the prevalence of smoking in male and female was 17.5% and 13.5% respectively [20]. This huge discrepancy between the gender-based prevalence could contribute to differences in the prevalence of positive smoker identity.

Males were more likely to have positive smoker identity compared to female, according to Tombor et al. [1]. Furthermore, an increase in social motives was associated with smoker identity development among males [21]. The meaning of social motive was that behaviors which brought about positive perception of oneself by others would be integrated into one’s identity. On the basis of this hypothesis, being in a high-smoking-prevalence environment such as Kelantan would definitely strengthen positive smoker identity among male smokers because the male smokers kept on enhancing each other’s positive viewpoints towards smoking. In addition, there was a relationship between the extent of gender difference in social perceptions of cigarette smokers and the extent of gender difference in smoking prevalence [22]. This observation could also probably be true in Kelantan where most the population were conservative Muslim Malays. Female smokers were probably more negatively evaluated in terms of health, purity, respect, self-control and good judgment here compared to Kuala Lumpur or other cities in the west coast of Peninsular Malaysia, where there were mixed-race populations. So, females in Kelantan were less likely to smoke due shared bad perceptions on female smokers, which possibly contributed to low positive smoker identity among the female smokers and its prevalence.

A significant association between SSCM-M and positive smoker identity was a further proof concurrent validity between the two scales. It illustrated that both had possibly share a similar theoretical groundwork, although they were different questionnaires with different items. Shadel and Mermelstein [10] demonstrated that smokers with a low self-concept had stronger odds of being abstinent after 3 months. This finding proposed that labeling—or considering one’s self as a non-smoker—was crucial to quitting success. A significant association between heaviness index and positive smoker identity that we found in our study was consistent with the findings by Tombor et al. [1], Levinson et al. [6] and van den Putte et al. [14]. The association was simply sensible because a smoker with positive smoker identity would definitely love cigarettes. This love or affinity towards cigarette would be translated into the real action or habit of smoking, in term of its frequency and amount.

Higher education attainment (certificate holder or higher) was found to be inversely associated with positive smoker identity in our study. Smokers who were a certificate holder or higher-education-level holder had 0.46 times chances to have positive smoker identity compared to smokers who had lower education attainment given other confounders were adjusted. This result demonstrated the protective effect of educational attainment against positive smoker identity. Gilman et al. [23] reported that lower education was associated with more pack-years of smoking, fewer quit attempts and a lower likelihood of cessation, after controlling for measured confounders. This finding was also supported by Koning et al. [24] who observed that one additional year of education caused 9 months duration of smoking reduction and 4.4% decrease in the probability of being a current smoker [25]. However, Maralani [26] discovered it was not as straightforward as that. They reported prediction of smoking in adulthood were based on decisions made during adolescence. They found that school policies, peers and youths’ mortality expectations predicted smoking in adulthood, but that college aspirations and analytical skills did not.

There were a number of limitations of this study. First, the participants recruited for this research were all adult males from the urban population whose behavior and demeanor may not be the representative of the female smoking population. Secondly, self-reported data on smoking and other variables may not be accurate enough due to recall bias and social desirability bias. Another limitation of the study was that an attrition rate of almost 20% from the sample pool and failure to recruit more samples to fulfill sample size requirement may contribute to volunteer bias. However, we minimized this bias was by making sure the data collection session a subtle process, whereby the invitation letters sent to their bosses were written in a clear yet understated manner. Also, the slot for data collection was also carried out in an informal way during a bigger event where health education lectures and anti-tobacco campaigns were delivered.

## 5. Conclusions

The prevalence of smokers with positive smoker identity among 253 workers in federal and local agencies in Kota Bharu was 72.3% (95% CI: 67–78%). The final model in multiple logistic regression showed 4 significant factors after all the other confounders controlled—older age, higher SSCS-M score, higher heaviness index and lower education attainment level. This finding recommended smoking cessation effort such as cessation promotion and enrollment into smoking clinics should be prioritized and targeted at those who are younger and who have higher educational attainment. The prioritization would lead to best interventional outcomes and better use of limited resources. Apart from that, the prevalence of PSI would provide an objective indicator for tobacco denormalization status in a population. All the stakeholders in relation to WHO tobacco endgame strategies should take the opportunity to utilize the availability of this pioneering instrument in measuring the denormalization of smoking behavior indicator, which is one of four main indicators in realizing the tobacco endgame ambition.

This study was not without its limitations. Further studies on different population such as adolescent or female smokers or in more suburban or rural areas would be recommended. Future studies with bigger sample size and more diverse variables would probably unearth many more predictors strongly correlated and significantly associated with positive smoker identity. Longitudinal studies monitoring the trend in a specified population or interventional studies observing the change of positive smoker identity after health promotion programs, smoking cessation treatments, smoking-related policy changes or any other interventions, would be excellent in fully utilizing the potential of PSmoQi. Time-trend analysis and comparison of positive smoker identity prevalence among communities, states, countries and regions worldwide would provide huge insights in what to focus on, how much work to do and how to improve the denormalization of smoking culture, which would also mean further curtailing positive smoker identity in our humanity.

## Figures and Tables

**Table 1:** Prevalence of positive smoker identity (PSI), or constructs identical to it, and linked, correlated or associated factors.

Studies	Population	Prevalence	Factors
Berg et al., 2009 [4] Minnesota, USA	College students	49.3%	Older Female Attended 2-year (versus 4-year) college No alcohol consumption in last 30 days More attempts to quit
Choi et al., 2010 [5] Michigan, USA	University students	26.2%	Smoked everywhere in all situations Smoked while driving Bought cigarette for themselves Smoked more number of cigarettes in last 30 days Senior students (versus freshmen) Had more negative affect reduction Had more social facilitation More smokers in their social network Felt more peer pressure to quit smoking Felt more peer pressure to modify smoking behavior
Levinson et al., 2007 [6] Denver, USA	College students	43.7%	More frequent smoking Increased smoking after entering college Most close friends were smokers Wanted to quit smoking More addicted to smoking Smoked when drinking More failed attempts Preferred to date smokers Did not advocate tobacco-free campus
Ridner et al., 2010 [7] Kentucky, USA	College students	33.1%	Higher smoking rate More frequent smoking
Hertel and Mermelstein, 2012 [8] Chicago, USA	High school students	Not documented	Smoking escalation
Falomir and Invernizzi, 1999 [9] Spain	Secondary school students	Not documented	Smoking behavior Decreased intention to give up smoking Lack of behavioral control More number of cigarettes Longer duration of smoking Less intention to quit More motivated to cope with threat to their identity Overestimated social support on behavior
Shadel and Mermelstein, 1996 [10] Chicago, USA	Clinic-based smoking cessation program adult clients	Not documented	Cessation failure Lower chance of being abstinent
Tombor et al., 2013 [1] UK	National adult survey	18.3%	Older Male Stronger nicotine dependence Lower motivation to stop smoking Not having made quit attempt in the past year Enjoyment of smoking Addiction to smoking Lower confidence in ability to quit smoking No current and future health concern No concern about effect of smoking on family Higher cost of smoking Less quit attempts
Tombor et al., 2015 [11] UK	Adult household survey	19.7%	Older Shorter duration of abstinence Needed aids for quitting

**Table 2:** Sample size calculation for the objective of determining factors associated with positive smoker identity.

Factor	α	Power	P0	P1	m	Sample Size
Male [5]	0.05	0.8	0.14	0.30	1	208
Nicotine Dependent [1]	0.05	0.8	0.26	0.40	1	352
Low Motivation to Stop [1]	0.05	0.8	0.23	0.38	1	294

**Table 3:** Socio-demographic characteristics of the participants (*n* = 253).

Variable	*n* (%)
Median age (inter-quartile range)	40 (14.00)
Sex	
Men	253 (100)
Women	0 (0)
Ethnicity	
Malay	253 (100)
Others	0 (0)
Education level	
Secondary school or lower	132 (52.2)
Certificate or Diploma Level	96 (37.9)
Bachelor’s degree or higher	25 (9.9)
Job level	
Lower staff	177 (70.0)
Middle manager	69 (27.3)
Top manager	7 (2.8)
Marriage Status	
Single	24 (9.5)
Married	225 (88.9)
Divorced	4 (1.6)
Median Income (Ringgit Malaysia(RM)) (inter-quartile range)	RM2500 (1335)

**Table 4:** Smoking behavior, cigarette cessation behavior, self-reported health status and co-morbidities, their awareness towards anti-smoking campaigns and the economics of smoking (*n* = 253).

Variable	*n* (%)
Smoker type	
Daily	189 (74.7)
Occasional	64 (25.3)
Tobacco products consumed	
Conventional cigarette	244 (96.4)
Vape	16 (6.3)
Shisha	1 (0.4)
Pipe	3 (1.2)
E-cig	1 (0.4)
Others	9 (3.6)
Median age start smoking (inter-quartile range)	18 (5.00)
Frequency of smoking	
Daily	223 (88.1)
Once a week	10 (4.0)
Once a month	2 (0.8)
Less frequent than once a month	18 (7.1)
No. of cigarette per day	
1 or less	21 (8.3)
2 to 5	62 (24.5)
6 to 10	61 (24.1)
11 to 20	83 (32.8)
More than 20	26 (10.3)
No. of days smoked per month (last month)	
0 days	8 (3.2)
1–2 days	4 (1.6)
3–5 days	17 (6.7)
6–9 days	4 (1.6)
10–19 days	27 (10.7)
20–29 days	29 (11.5)
Full 30 days	164 (64.8)
Place of smoking	
Home	172 (68.0)
Workplace	142 (56.1)
Friend’s house	74 (29.2)
Food café	171 (67.6)
Public place	59 (23.3)
Social gathering	81 (32.0)
Others	24 (9.5)
Ways of getting cigarettes	
Shop	230 (90.9)
From friends	53 (20.9)
Stole it	2 (0.8)
Others buy it for me	6 (2.4)
Other ways	1 (0.4)
Mean number of cessation trial in the last 1 year (SD)	1.2 (2.20)
Methods of smoking cessation trial	
Never stop	98 (38.7)
Willpower	129 (51.0)
Over-the-counter medications	17 (6.7)
Quitline	1 (0.4)
Friends’ assistance	13 (5.1)
Counselling by HCW	14 (5.5)
Professional NRT	5 (2.0)
Others	14 (5.5)
Self-reported health status	
Very good	31 (12.3)
Good	211 (83.4)
Poor or very bad	11 (4.3)
Presence of co-morbidity	
Asthma	12 (4.7)
COPD	1 (0.4)
Hypertension	30 (11.9)
Diabetes Mellitus	19 (7.5)
Hypercholesterolemia	25 (9.9)
Other diseases	10 (4.0)
Exposure to smoking cessation campaign	
Often	87 (34.4)
Occasional	132 (52.2)
Never	34 (13.4)
Median cost of smoking per month (interquartile range)	RM120 (130)
Usage of cheaper than market price cigarette	
All of them (100%)	66 (26.1)
Most of them (70 to 99%)	74 (29.2)
Occasionally (30 to 69%)	48 (19.0)
Rarely (1 to 29%)	23 (9.1)
Never	42 (16.6)

HCW-Healthcare worker, NRT—Nicotine replacement therapist, COPD—Chronic Obstructive Pulmonary Disease.

**Table 5:** Factors associated with positive smoker identity using simple logistic regression.

Factors	Crude OR (95% CI)	Wald Stat	*p*-Value	Selected for Multiple Logistic Regression
Categorical				
Smoking Status (Occasional)	0.54 (0.29, 0.98)	4.07	0.044	Yes
Education (Certificate or higher)	0.43 (0.24, 0.76)	8.56	0.003	Yes
Job level				
Low	1			Yes
Middle	0.58 (0.32, 1.06)	3.15	0.076	
Top	1.99 (0.23, 16.94)	0.39	0.531	
Marriage status				
Married	1	0.10	0.755	No
Single	1.17 (0.44, 3.07)			
Divorced	1.17 (0.12, 11.43)	0.02	0.895	
No of day cig smoked last 30 days				
Full 30 days				
Zero	1			No
1–2 days	0.08 (0.02, 0.42)	8.97	0.003	
3–5 days	0.73 (0.07, 7.22)	0.07	0.786	
6–9 days	0.35 (0.12, 0.98)	3.99	0.046	
10–19 days	0.24 (0.03, 1.79)	1.93	0.164	
20–29 days	0.40 (0.17, 0.92)	4.61	0.032	
No of cigs per day				
11 to 20	1			No
Less than 1	0.13 (0.04, 0.48)	9.55	0.002	
1	2.08 (0.24, 17.97)	0.44	0.506	
2 to 5	0.44 (0.21, 0.90)	5.00	0.025	
6 to 10	1.21 (0.54, 2.73)	0.22	0.643	
More than 20	1.63 (0.50, 5.33)	0.66	0.416	
Conventional Cig	3.44 (0.90, 13.21)	3.24	0.072	Yes
Vape	0.27 (0.10, 0.76)	6.23	0.013	Yes
Bought cigs at shops	4.83 (1.99, 11.77)	12.04	0.001	Yes
Got cigs from friends	0.76 (0.40, 1.47)	0.65	0.421	No
Smoked at home	1.64 (0.92, 2.91)	2.81	0.094	Yes
Smoked at workplace	1.20 (0.69, 2.09)	0.42	0.517	No
Smoked at friend’s house	2.19 (1.12, 4.31)	5.17	0.023	Yes
Smoked at food café	1.74 (0.98, 3.08)	3.55	0.059	Yes
Smoked at public place	2.19 (1.04, 4.62)	4.27	0.039	Yes
Smoked at social gathering	1.86 (0.99, 3.51)	3.67	0.056	Yes
Used willpower to stop	0.43 (0.24, 0.77)	8.20	0.004	No
Used OTC medication to stop	1.85 (0.51, 6.65)	0.89	0.346	No
Sought friend’s help to stop	4.84 (0.62, 37.96)	2.25	0.133	No
Sought health counselling	1.43 (0.39, 5.28)	0.29	0.593	No
Sought professional NRT	1.54 (0.17, 14.04)	0.15	0.701	No
Self-reported health				
Good	1			No
Very good	0.67 (0.30, 1.49)	0.95	0.329	
Poor	1.67 (0.35, 7.94)	0.41	0.522	
Had asthma	1.15 (0.30, 4.40)	0.05	0.832	No
Had hypertension	3.87 (1.13, 13.18)	4.67	0.031	Yes
Had diabetes mellitus	1.47 (0.47, 4.60)	0.44	0.505	No
Had hypercholesterolemia	0.98 (0.39, 2.46)	0.00	0.969	No
Watched stop smoking campaigns (often)	0.78 (0.44, 1.38)	0.75	0.387	Yes
Had below market value cigs				
All 100%	1			Yes
Most of them (70–99%)	0.84 (0.38, 1.85)	0.19	0.662	
Sometimes (30–69%)	0.59 (0.25, 1.38)	1.46	0.227	
Rarely (1–29%)	0.51 (0.18, 1.43)	1.66	0.198	
Never	0.49 (0.20, 1.15)	2.70	0.100	
Continuous Factors				
Income (Ringgit Malaysia; RM)	1.00 (1.00, 1.00)	0.12	0.730	No
Age (years)	1.05 (1.02, 1.08)	8.35	0.004	Yes
Age first smoked (years)	0.98 (0.93, 1.03)	0.66	0.415	No
Smoking heaviness index	1.00 (1.00, 1.00)	11.52	0.001	Yes
No of stop attempt	0.87 (0.77, 0.98)	5.46	0.019	Yes
Smoking cost	1.00 (1.00, 1.00)	7.81	0.005	Yes
FTND-M Score	1.18 (1.03, 1.35)	5.97	0.015	Yes
CSEQ-M Score	0.98 (0.96, 0.99)	5.94	0.015	Yes
SSCS-M Score	1.20 (1.11, 1.29)	22.80	<0.001	Yes

Note: FTND—M (Fagerstrom test of nicotine dependence (Malay Version); CSEQ—M (Cessation self-efficacy questionnaire (Malay version); SSCS-M (Smoking self-concept scale (Malay version).

**Table 6:** Factors associated with positive smoker identity using multiple logistic regression.

Variables		Crude OR ^a^ (95% CI)	Adjusted OR ^b^ (95% CI)	Wald Stat ^b^ (df)	*p*-Value ^b^
Age		1.055 (1.021, 1.089)	1.042 (1.004, 1.081)	4.81 (1)	0.028
SSCS-M Score		1.198 (1.109, 1.293)	1.216 (1.112, 1.329)	18.31 (1)	<0.001
Heaviness index		1.003 (1.001, 1.004)	1.002 (1.001, 1.004)	6.53 (1)	0.011
Education attainment					
(Certificate or higher)	No	1.000	1.000		
	Yes	0.414 (0.229, 0.746)	0.458 (0.233, 0.900)	5.13 (1)	0.024

^a^ Simple logistic regression; ^b^ Multiple Logistic Regression model is applied. Multi-collinearity and interaction term were checked and not found. Hosmer-Lemeshow test (*p* = 0.546), classification table (overall correctly classified percentage = 76.5%) and area under ROC curve (72.0%) were applied to check the model fitness.
